# Case Report: Severe vitamin D deficiency in a girl with inflammatory myopathy and myonecrosis

**DOI:** 10.3389/fped.2024.1339875

**Published:** 2024-02-12

**Authors:** Andrew Kanouse, Benjamin Udoka Nwosu, Parissa Salemi

**Affiliations:** Division of Endocrinology and Metabolism, Department of Pediatrics, Cohen Children’s Medical Center, New Hyde Park, NY, United States

**Keywords:** vitamin D deficiency, myonecrosis, myopathy, case report, hypovitaminosis D

## Abstract

Inflammatory myopathies are a rare group of disorders that can cause significant disruption in the ability of an individual to adequately perform activities of daily living. In this case report, we present a case of a girl presenting with a substantial compromise of her ambulation with a muscle biopsy consistent with myonecrosis. She was subsequently diagnosed with an inflammatory myopathy and started on glucocorticoid and methotrexate therapy with minimal symptomatic improvement. Further in her clinical course, hypocalcemia and an undetectable 25-hydroxyvitamin D level were detected. Prompt institution of calcium and vitamin D supplementation significantly improved her myopathic condition. While there is evidence in the literature linking vitamin D deficiency with myopathy, there is a lack of data on the association between hypocalcemia and vitamin D deficiency with myonecrosis, which could represent comorbid states in myonecrosis. Therefore, vitamin D status should be established in all patients with myonecrosis, as vitamin D deficiency is easy to diagnose and treat, as exemplified in our patient’s case, which shows that such treatment could lead to significant clinical improvement.

## Introduction

Inflammatory myopathies are a rare group of disorders that present with muscle weakness and pain, elevated inflammatory markers and muscle enzymes, and, if performed, muscle biopsy findings consistent with inflammation. A less commonly considered cause of myopathy is vitamin D deficiency (1). The impact of nutrient deficits such as vitamin D deficiency on the etiopathogenesis and the clinical course of myopathy is not clear. Vitamin D deficiency, defined as a serum 25-hydroxyvitamin D [25(OH)D] concentration of <20 ng/ml (<50 nmol/L), is not uncommon, with an estimated 1 billion individuals worldwide either having deficiency or insufficiency (2). Reports on individuals diagnosed with myopathy and coexisting vitamin D deficiency noted a clinical improvement in symptoms following vitamin D supplementation (3). These myopathies have been associated with the findings of atrophic muscle fibers on biopsy. In this study, we present a case of a 17-year-old girl with prolonged worsening mobility, who was diagnosed with inflammatory myopathy and myonecrosis in a setting of severe vitamin D deficiency, with symptom improvement noted following vitamin D and calcium supplementation.

## Case report

The patient was a non-verbal 17-year-old girl with autism spectrum disorder, global developmental delay, and iron-deficiency anemia, who was referred to the Orthopedic Surgery Department after she suffered from worsening gait for 4–5 months. Her parents reported that she had reduced muscle tone at birth, which was attributed to a premature gestation of 29 weeks. Later, her hypotonia relieved with the initiation of occupational therapy.

Her symptoms of weakness initially started with difficulty in climbing stairs and with other activities of daily living, such as toileting, bathing, and dressing herself. Her gait was described as “extremely slow,” with a more pronounced weakness in the legs than in the arms and “worse” on the left side compared with the right side of her body. A knee x-ray, obtained by orthopedic surgery, demonstrated a lucency without a clear etiology. Her neurology consult in the emergency room assessed her muscle weakness to be late-onset cerebral palsy from prematurity. The result of a fresh x-ray taken at her subsequent visit to the Orthopedic Department was read as normal. However, an evaluation done at the neurology clinic around the same time revealed a mild foot drop, inability to rise from a chair without the use of the arms, inability to flex the hip while sitting, and absent deep tendon reflexes. Her laboratory workup showed a creatinine kinase (CK) level of 1,972 U/L (normal range 25–170 U/L), with a confirmatory repeat level of 1,748 U/L, yielding a preliminary diagnosis of inflammatory myopathy. The following month, she was examined by physicians at the Physical Medicine and Rehabilitation Department, who noted significant extremity contractures and muscular stiffness; she was then given an adaptive stroller.

## Diagnostic assessment and treatment

**Figure 1:**
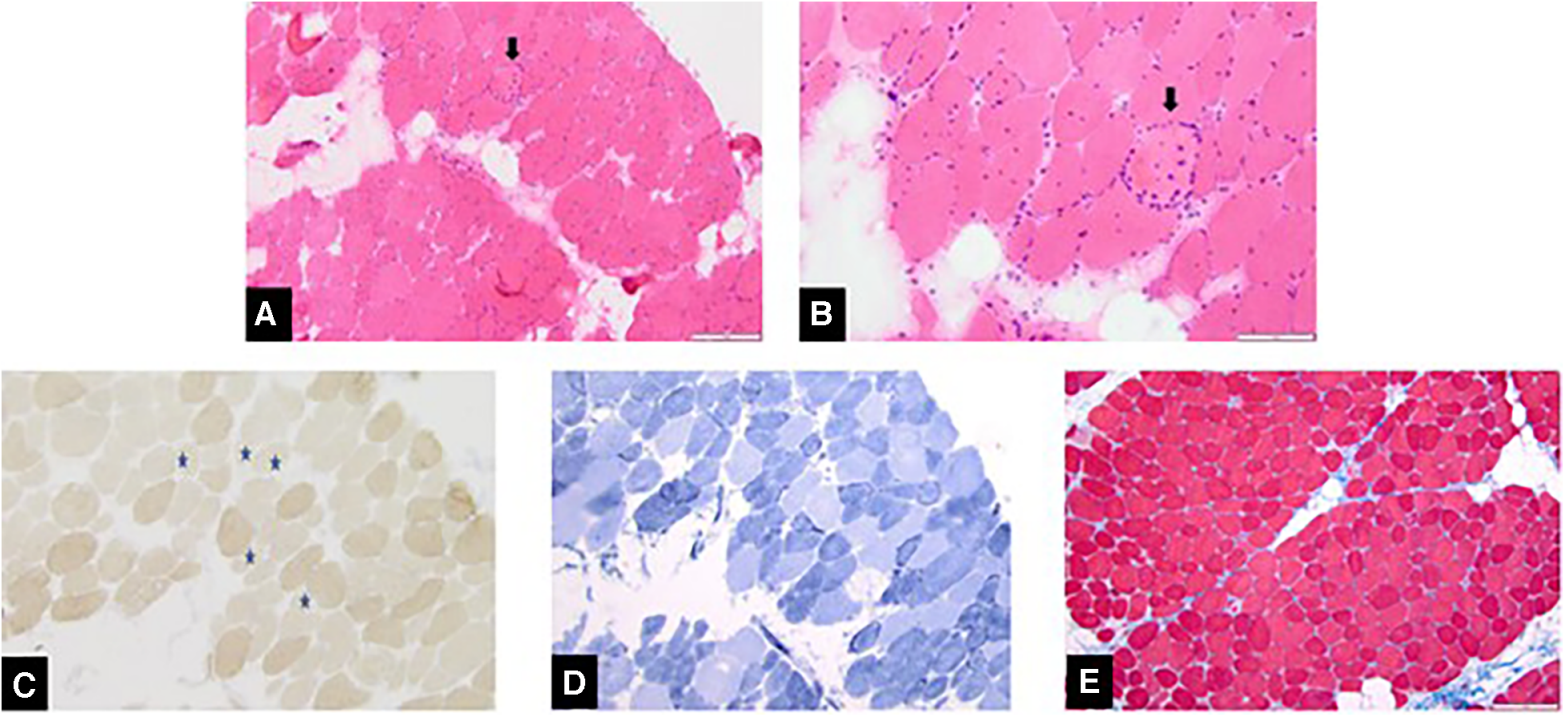
Histologic panels of muscle biopsy showing (**A**) variable muscle fiber size due to an atrophy of numerous muscle fibers. (**B**) In a magnified image, the arrow denotes a necrotic fiber with mononuclear inflammatory cells. (**C**) The asterisks indicate type 1 fibers, which are noted to be the predominant fiber type undergoing atrophy. (**D**) Scattered ring fibers, suggesting prolonged myopathy. (**E**) Endomysial fibrosis, also suggestive of chronic myopathy.

She subsequently underwent a nerve conduction study, the result of which was normal. Her genetic workup for muscular dystrophy, rhabdomyolysis, and metabolic myopathy demonstrated a variant of unknown significance that was thought to be benign (*SYNE2* and *TRMT5* genes). Her muscle biopsy revealed “features consistent with a chronic, active myopathy. Inflammatory response is largely associated with myonecrosis.” The description of her muscle fibers noted that her “variation of fiber size is significantly increased due to the presence of fairly numerous atrophic fibers varying in size, as well as a few hypertrophic fibers,” and that “the majority of atrophic fibers are type 1, with few mildly atrophic type 2 fibers” (Figure 1). As a result of these findings, her diagnosis was revised to immune-mediated necrotizing myopathy and she was referred to the Rheumatology Department for further investigation. Her rheumatological workup is summarized in Table 1. This workup revealed a total calcium level of 7.2 mg/dl (1.8 mmol/L, normal range 8.6–10.0 mg/dl) and an alkaline phosphatase level of 565 U/L. However, these abnormalities were left unexamined at the time. She was started on prednisolone (1.25 mg/kg daily) and methotrexate (4 mg once weekly), 10 months after her initial symptoms, as an anti-inflammatory and immunosuppressive regimen for the diagnosis of inflammatory myopathy. She experienced minimal to no improvement when she taking these medications for 4 months. Laboratory tests obtained 4 months into therapy showed a CK of 709 U/L and a total calcium level of 5.5 mg/dl (1.38 mmol/L; normal range 8.6–10.0 mg/dl). She was subsequently sent to the Emergency Department (ED) for the management of severe hypocalcemia.

**Table 1:** Summary of the patient's baseline laboratory data.

Parameters	Results
Aldolase	18.9 U/L (high, ref 3.3–10.3)
Von Willebrand factor antigen	119% (normal)
C-reactive protein (CRP)	<3 mg/L (normal)
Creatine phosphokinase (CPK)	1,884 U/L (high, ref 25–170)
Lactate dehydrogenase (LDH)	446 U/L (high, ref 50–242)
Carbon dioxide (CO_2_)	20 mmol/L (slightly low, ref 22–31); 20 mEq/L
Blood urea nitrogen (BUN)	4 mg/dl (low, ref 7–23); 1.4 mmol/L
Creatinine	0.40 mg/dl (low, ref 0.5–1.3); 35 μmol/L
Albumin	3.7 g/dl (normal); 37 g/L
Calcium	7.2 mg/dl (low, ref 8.4–10.5); 1.8 mmol/L
Alkaline phosphatase	565 U/L (high, ref 30–120 U/L)
Myomarker panel 3: anti-Jo-1 Ab, PL-7, PL-12, EJ, OJ, SRP, MI-2, TIF GAMMA (P155/140), MDA-5 (P140) (CADM-140), NPX-2 (P140), anti-PM/Sci-100 Ab, fibrillarin (U3 RNP), U2 snRNP, anti-U1-RNP Ab, KU, anti-SS-A 52 kD Ab, IgG	All negative except for anti-SS-A 52 kg Ab IgG: 35 u (ref <20)

In the ED, a physical examination was done and she tested negative for tremors, spasms, paresthesia, numbness, or tetany. Further laboratory studies showed that she was hypocalcemic with a total calcium level of 4.8 mg/dl (1.2 mmol/L) and an ionized calcium level of 2 mg/dl (0.5 mmol/L; normal range 4.64–5.27 mg/dl). She was also hypophosphatemic (a phosphorus level of 2.4 mg/dl; 0.78 mmol/L; normal range 2.7–4.5 mg/dl) and hypomagnesemic (a magnesium level of 0.3 mEq/L; 0.62 mmol/L; normal range 1.3–2.0 mEq/L). Her parathyroid hormone level was elevated at 496 pg/ml (496 ng/L; normal range 15–65 pg/ml). An ECG revealed a prolonged QTc of 515 ms (normal 360–460 ms), with a sinus rhythm and associated first-degree atrioventricular block. She received several boluses of intravenous calcium gluconate infusions, as well as oral calcium carbonate, cholecalciferol, and calcitriol, which alleviated her calcemia.

Her pretreatment serum 25(OH)D level obtained at presentation, i.e., 13 months following her initial symptoms, showed an undetectable 25(OH)D of <5.0 ng/ml (<12.5 nmol/L; normal range 30–80 ng/ml) and a low 1,25-dihydroxyvitamin D of 14.8 pg/ml (35.5 pmol/L; normal range 19.9–79.3 pg/ml), which were consistent with severe vitamin D deficiency. Upon further review, her parents confirmed that the patient was on a Caribbean diet, which consists mainly of fruits, vegetables, rice, and legumes. She did not consume dairy products, and overall, her diet was low in calcium and vitamin D.

**Figure 2:**
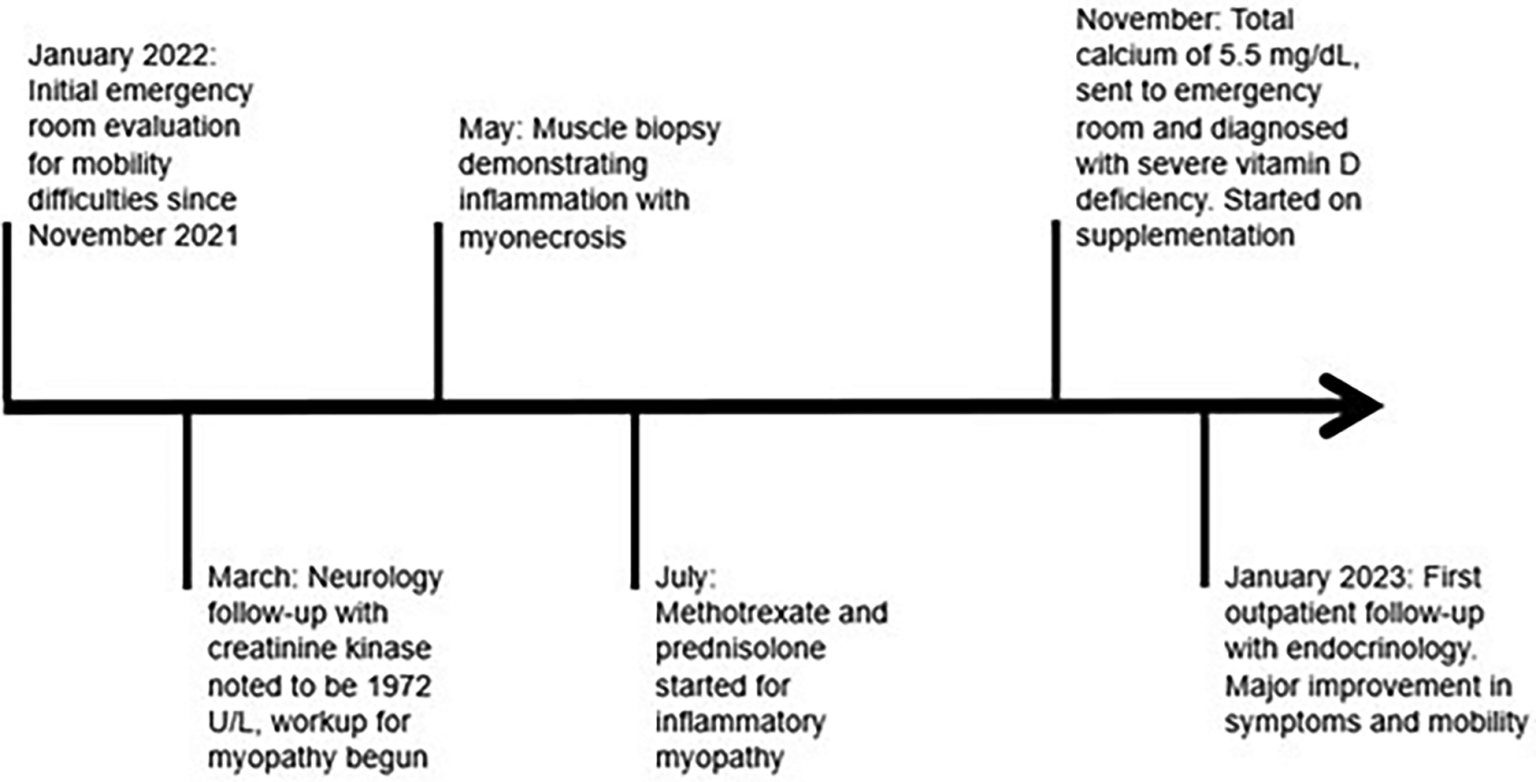
A chronological representation of the patient's clinical course with associated medical interventions.

She spent three days in the hospital and received eight intravenous boluses of calcium gluconate (1,750–2,000 mg each), one potassium chloride bolus, and one magnesium sulfate bolus. At the time of discharge, her serum calcium level was 8.1 mg/dl (2.0 mmol/L). She was discharged on 70 mg/kg/day of elemental calcium, 8,000 international units of cholecalciferol daily, and 0.1 μg of calcitriol twice daily, and methotrexate was discontinued. Her clinical timeline is shown in Figure 2.

## Outcome and follow-up

Her serum calcium levels remained in the normal range with calcium and vitamin D supplementation after discharge. Within one month of discharge, there was a significant improvement in her muscle strength, as she was able to ambulate, rise from a chair, and transfer herself to and from her bed without assistance. She was unable to accomplish any of these activities in the months preceding her vitamin D and calcium supplementation. She continued her pre-existing corticosteroid for 6 weeks after her discharge. At her 2-month postdischarge visit, her gait was normal and without a limp. Her clinical and biochemical profiles continued to improve on calcium and vitamin D supplementation (Table 2), and by the time of her 3-month visit, her CK level had normalized, her muscle weakness had nearly resolved, and she could ambulate easily.

**Table 2:** A longitudinal profile of the patient's mineral metabolic laboratory parameters.

Laboratory evaluation	At presentation to emergency room	At discharge from hospital	1 month later	3.5 months later	Reference range in conventional units
Ionized calcium	2 mg/dl (0.5 mmol/L)	3.12 (0.78)			4.8–5.52 mg/dl
Total calcium	4.8 mg/dl (1.2 mmol/L)	8.1 (2.0)	8.6 (2.2)	9.2 (2.3)	8.6–10.0 mg/dl
Phosphorus	1.7 mg/dl (0.55 mmol/L)	2.6 (0.8)			2.7–4.5 mg/dl
Magnesium	1.5 mg/dl (0.6 mmol/L)	2.1 (0.86)		1.9 (0.78)	1.3–2.0 mg/dl
Parathyroid hormone	496 pg/ml (496 ng/L)		254 (254)	8 (8)	15–65 pg/ml
25-hydroxyvitamin D	<5 ng/ml (<12.5 nmol/L)		13.8 (34.5)	31.9 (79.8)	30–80 ng/ml
1, 25-dihydroxyvitamin D	14.8 pg/ml (35.5 pmol/L)				19.9–79.3 pg/ml

## Discussion

Myopathy secondary to vitamin D deficiency is described in adult case reports, but less so in the pediatric population. In adults, myopathy secondary to vitamin D deficiency is suspected to be a presenting feature in 30% of patients diagnosed with osteomalacia, the most common cause of which is vitamin D deficiency (4). Muscle weakness may be non-specific, but in adolescents, it is often proximal, as seen with difficulties in rising from a seated position, ascending stairs, and extending the knees, with associated abnormal gait (5). Similar to our patient case, Prabhala et al. describe the cases of five adult patients with a significant decline in mobility, even requiring wheelchairs, receiving alternative diagnoses with unsuccessful treatments prior to a diagnosis of vitamin D deficiency (6). Supplementation led to a rapid alleviation of symptoms. Fabbriciani et al. describe the case of a 20-year-old man with muscle and gait disturbances with an undetectable vitamin D concentration that was believed to be attributed to vegetarianism and poor sunlight exposure. Following supplementation, his gait disturbances resolved (7). These cases are slowly progressive in nature and therefore can be easily overlooked. In a study of 47 adolescent and adult women by Al-Said et al., it was found that the onset of symptoms spanned a period of >1 year, leading to an initial consideration of other differential diagnosis such as orthopedic disease, psychopathology, and inherited myopathies (4).

Vitamin D deficiency is an overlooked but common disease condition, especially in those with risk factors such as age (elderly or infancy), dark complexion, minimal sunlight exposure, particular altitudes/distance from the equator, medications that alter vitamin D absorption, biliary disease, malabsorption, pancreatic disease, renal disease, limited diet, and liver disease.

Reports suggest that vitamin D deficiency impairs muscle function, but the mechanism is unclear (5). It is hypothesized that vitamin D deficiency induces reduced calcium influx into the sarcoplasmic reticulum of the skeletal muscle and associated malfunction of the vitamin D receptors (VDR), which leads to decreased concentrations of intracellular calcium that is needed for optimal contractility (8). The importance of such receptors has been demonstrated by an alteration of quadriceps and grip strength corresponding to the polymorphisms of VDR expressed in muscle tissue (9). Specifically, there is an association between VDR and fiber size (1). Consequently, vitamin D deficiency and resultant hypocalcemia, as in our patient, could result in subsequent muscle weakness. Therefore, reduced calcium influx into the sarcoplasmic reticulum may contribute to the mechanism associated with muscle remodeling and myonecrosis, which could be ameliorated with vitamin D supplementation. It is also possible that vitamin D has a genomic effect on calcium uptake into cells, transport of phosphate, muscle fiber differentiation, and protein synthesis, which could also lead to myonecrosis (1). The association between vitamin D deficiency and various autoimmune diseases makes it plausible to consider an association between vitamin D deficiency and inflammatory myopathies (10).

Our patient had prolonged and severe vitamin D deficiency, given her undetectable serum 25(OH)D, and subsequent significant hypocalcemia. Typically, biopsies of myopathies secondary to vitamin D deficiency are non-specific but commonly demonstrate atrophic fibers, especially type II fibers (4, 5). Often, there is also a paucity of inflammation or identifiable primary muscle disease (3, 7). Usually, there is a propensity for enlargement of the spaces between muscle fibers secondary to glycogen, fat, or fibrosis (5). All these features were identified histologically in the biopsy of our patient but with a unique component of myonecrosis. In our review of the literature, we found that myonecrosis is not usually associated with vitamin D deficiency (3). However, many prior case reports did not study patients with undetectable serum 25(OH)D, as in our patient, and this subsequently resulted in a prolonged time period with an ongoing depletion of calcium stores, which is also likely to have contributed to such an extreme presentation. Therefore, this unique histopathological finding of myonecrosis may be associated with severe underlying vitamin D deficiency, coupled with chronic hypocalcemia, which resulted in the extreme compromise of mobility. While the precise etiology of the myonecrosis remains unclear, the hypocalcemia and vitamin D deficiency appear to be strongly associated with the patient's clinical presentation and should be considered a potential association with the development of myonecrosis as well.

Vitamin D deficiency should be considered an associated factor when myonecrosis is noted on pathology in patients with inflammatory myopathy. Given the prevalence of vitamin D deficiency in the pediatric population and the ease of treatment, it is important to evaluate vitamin D status in all patients who present with suboptimal mobility secondary to myopathy, especially in those identified with myonecrosis, and, if deficiency is found, vitamin D supplementation should be initiated immediately.

## Data Availability

The original contributions presented in the study are included in the article/Supplementary Material, and further inquiries can be directed to the corresponding author.
